# Sequential Production of Levulinic Acid and Porous Carbon Material from Cellulose

**DOI:** 10.3390/ma11081408

**Published:** 2018-08-11

**Authors:** Shimin Kang, Jiaming Pan, Guoting Gu, Chong Wang, Zepan Wang, Jionghao Tan, Guiheng Liu

**Affiliations:** 1 Guangdong Provincial Key Laboratory of Distributed Energy Systems, Dongguan University of Technology, Dongguan 523808, China; kangshimin@dgut.edu.cn (S.K.); m13559770641_1@163.com (J.P.); 13559772574@163.com (G.G.); zepanwang@163.com (Z.W.); KingHowe_Tam@163.com (J.T.); as_9301@163.com (G.L.); 2 College of Mechanical Engineering, Dongguan University of Technology, Dongguan 523808, China; 3 Engineering Research Center of None-food Biomass Efficient Pyrolysis and Utilization Technology of Guangdong Higher Education Institutes, Dongguan University of Technology, Donguan 523808, China

**Keywords:** cellulose, levulinic acid, porous carbon material, pyrolysis, hydrolysis, adsorption

## Abstract

A sequential production of levulinic acid (LA) and porous carbon material (CM) from cellulose was conducted by a two-step process. The cellulose was first acid hydrolyzed, and the preferred reaction conditions required a severity factor of 4.0–4.5, in which the yields of LA, formic acid, and solid residue were 38 ± 3 wt%, 17 ± 3 wt%, and 15 ± 3 wt%, respectively. The solid residue was further used for CM preparation through pyrolysis, with or without ZnCl_2_ activation. The ZnCl_2_ activation promoted the formation of CMs with improved thermal stability, high surface area (1184–2510 m^2^/g), and excellent phenol adsorption capacity (136–172 mg/g). The used CM can be easily regenerated by a simple methanol Soxhlet extraction process, and a comparable phenol adsorption capacity of 97 mg/g was maintained for the 5th reusing. Finally, 100 g cellulose produced 40.5 g LA, 18.9 g formic acid and 8.5 g porous CM, with a total carbon utilization ratio reaching 74.4%.

## 1. Introduction

With increased concerns over sustainable economic growth and climate change, research on value-added chemicals and materials production from renewable resources has attracted much attention. Levulinic acid (LA) is a platform chemical that can be used to produce various chemicals, including plasticizers, fuel additives, herbicides, polymers, etc. [1,2,3,4] LA has also been used as a desirable feedstock for liquid fuel production, which was regarded as a chemical bridge connecting biomass and petroleum processing. [4,5] LA is currently primarily produced by maleic anhydride [2,6], which is costly and leads to weak industrial application. The price of LA was reported to be USD 5–8 per kg, and a target price of less than USD 1 per kg might be achieved through acid hydrolysis of cheap renewable sources [7]. The routine conversion of lignocellulose biomass into LA involves consecutive reactions, including hydrolysis of cellulose to glucose, dehydration of glucose into 5-hydroxylmethylfurfural, and rehydration of 5-hydroxylmethylfurfural to LA and formic acid [4,8,9]. Here, formic acid is a valuable product that can be utilized for the synthesis of rubber, textiles, plasticizers, pharmaceutical products, etc. [2,4] Glucose and 5-hydroxylmethylfurfural are two universally acknowledged key intermediates [1,2,3,4]. However, both intermediates form solid byproducts, mainly humins in acid hydrolysis conditions. It has been reported that formation of humins was inevitable in the viewpoints of thermodynamic characteristics of acid-catalyzed hydrolysis, and a high initial reactant concentration caused a high yield of humins [4,10,11]. Typically, the humins yields reached 21–36 wt% and 18–23 wt% from hydrolysis of glucose and 5-hydroxylmethylfurfural, respectively [12,13,14,15]. The humins generally contained a carbon content of 55–65 wt% [4], which indicated that about 29–58% and 17–26% of the initial carbon in glucose and 5-hydroxylmethylfurfural were wasted as solid residue, respectively. The formation of solid residue seriously decreases biomass utilization efficiency, and value-added application of solid residue is essential for the whole hydrolysis process. To make the solid residue profitable, several works have focused on the production of value-added products, e.g., thermochemical conversion of solid residue to obtain bio-oils and synthesis gas [16,17]. Owing to the carbon rich characteristics, a potential way is to convert the solid residue to value-added carbon material (CM), e.g., carbon adsorbents [18]. Carbon adsorbents have a wide range of applications in the industry and daily life, especially for wastewater treatment [19]. Usually, carbon adsorbents can be produced from carbon rich-in organics by pyrolysis after physical and chemical activation methods. To promote the formation of porous CM, several activating agents have been developed, in which ZnCl_2_ is one of the most used chemical activating agent [20,21,22]. In this work, a two-step reaction process was developed to obtain valuable LA, formic acid, and CM (instead of solid residue) in a sequential way, with the objective to maximize the carbon utilization ratio of cellulose.

## 2. Materials and Methods

### 2.1. Materials

Microcrystalline cellulose (catalogue number: 1420402), levulinic acid (LA, 98%; catalogue number: 245974), formic acid (98%; catalogue number: 299272), glucose (99%; catalogue number: G0048), and ZnCl_2_ (98%; catalogue number: 975486) were obtained from J & K Chem. Reagent, Beijing, China. H_2_SO_4_ (98%; batch number: 201741) was obtained from Dongguan Dongjiang Chemical Reagent Co., Ltd., Dongguan, Guangdong, China.

### 2.2. Hydrolysis for LA Production

Cellulose hydrolysis for LA production was catalyzed by 0.2 M H_2_SO_4_ solution (pH = 0.4) from 150 °C to 210 °C. Reactions were conducted in a small (20 mL) unstirred polytetrafluoroethylene (PTFE) reactor with a pressure limitation of 3 MPa. 0.5 g cellulose and 10 mL H_2_SO_4_ solution were added into the PTFE reactor and heated in an air-circulated oven to a preset temperature for about 60 min. The holding time started after 60 min heating and was recorded thereafter. After reaction, the products were separated into water solution and solid residue by vacuum filtration. The solid residue was dried at 100 °C for further use. The water solutions were diluted with water in 100 mL volumetric flasks, and analyzed by high performance liquid chromatograph (HPLC, Shimadzu, Kyoto, Japan) equipped with a C6H10 column (Sigma-Aldrich, St. Louis, MO, USA) as described before [8]. The column was eluted by diluted sulfuric acid solution (pH = 2.0) at 50 °C with a current speed of 0.7 mL/min. The glucose remaining in the solid residue was measured by method NREL/TP-510-42618 [23].

### 2.3. Preparation of CMs

Both the direct pyrolysis and pyrolysis after ZnCl_2_ activation were conducted for CM preparation from solid residue. In the ZnCl_2_ activation process, pre-weighted solid residue and ZnCl_2_ were mixed and stirred for 12 h. The slurry obtained was dried at 120 °C for 12 h in an oven, and then used for pyrolysis. The pyrolysis was conducted in a horizontal cylindrical furnace at 300–900 °C with nitrogen flow (100 mL/min) for 2 h, and the heating rate from the room temperature to preset temperature was 10 °C/in. The pyrolysis derived samples were washed with excess hot DI water (90 °C), until the Zn^2+^ concentration in the washing solution was ignorable, detected by atomic absorption spectroscopy (Z-2000, Hitachi, Ltd., Tokyo, Japan). The washed samples were further dried at 100 °C to obtain CM. The obtained CM from direct pyrolysis at X °C was labeled as CMX. To distinguish the CMX, the CM produced at Y °C with ZnCl_2_ activation (solid residue: ZnCl_2_ = a:b) was labeled as CMY (a:b).

### 2.4. Sample Characteristics

X-ray diffraction (XRD) analysis was tested using a Rigaku D/max-IIIA X-ray diffractometer (Rigaku Corporation, Tokyo, Japan), with a scanning 2-theta from 10° to 90°. All the samples were ground to powder before the XRD measurement. Thermogravimetric (TG) analysis was conducted under air by a Netzsch 209F3 (NETZSCH-Gerätebau GmbH, Serb, Germany). About 5 mg sample was placed in a Al_2_O_3_ sample pan and heated from 50 °C to 550 °C with a heating rate of 10 °C/min, under 20 mL/min N_2_ flow. The functional groups were analyzed by Fourier transform infrared spectroscopy (FT-IR) using a Tensor 27 (Bruker, Karlsruhe, Germany) by using KBr as an interference-free matrix [24]. The FTIR transmission analysis was conducted by a wavenumber range from 1000 to 4000 cm^−1^, with a scanning resolution of 4 cm^−1^. The surface morphology of the samples was measured by using a scanning electron microscopy (SEM) system (JEOL JSM-6701F, Tokyo, Japan). Pretreatment of the samples by plasma gold plated coating was conducted just before the SEM analysis. The Brunauer-Emmett-Teller (BET) surface area and total Barrett-Joyner-Halenda (BJH) pore volume were determined by low-temperature (77.4 K) nitrogen adsorption using a Micromeritics Instruments TriStar II (Micromeritics Instrument Corporation, Norcross, GA, USA). Before the analysis, 0.07–0.1 g samples were put into the sample tube, and a degassing process at 160 °C for 6 h was conducted. The total acidic and alkaline groups were determined by the Boehm titration method and phenolphthalein was used as an indicator [25]. The carbon content in the solid residue and CM400(1:2) were determined with an elemental analyzer (Elementar Vario EL cube, Elementar, Langenselbold, Germany) at Tianhe Chemical Technology Co., Ltd. (Jining, China).

### 2.5. Adsorption of Phenol

Adsorption experiment was conducted in 50 mL sealed bottles, where 30 mL of phenol solution (400 mg/L) and 0.05 g CM prepared were added in each bottle. The adsorption was conducted in an isothermal shaker at 200 rpm and 30 °C for 24 h. After the adsorption process, the CM and the solution were separated by a routine vacuum filtration apparatus containing Büchne flask and filter paper. The CM and the solution were recovered on filter paper and in a Büchne flask, respectively. The concentration of phenol remaining in the solutions was analyzed by a Specord^®^ 210 Plus UV-vis spectrophotometer at 269 nm. The adsorption capacity was calculated based on the mass decrease of the phenol in the solution and the CM mass (see Equation (1)).

(1) $$\begin{array}{l} \mathrm{Adsorption}\;\mathrm{capacity}\;\left(\frac{\mathrm{mg}}{g}\right)\\ =\frac{\left(\mathrm{initial}\;\mathrm{phenol}\;\mathrm{concentration}-\mathrm{remained}\;\mathrm{phenol}\;\mathrm{concentration}\right)\times \mathrm{volume}}{\mathrm{CM}\;\mathrm{mass}}\times 100\% \end{array}$$

Regeneration and reuse of CM400(1:2) for phenol adsorption were also conducted. The used CM400(1:2) was put in a 60 mL Soxhlet extractor, and then extracted by methanol for 8 h. The methanol volatilization temperature was kept at 80 °C. After the methanol extraction process, the recovered CM400(1:2) was oven dried at 60 °C for 2 h to obtain the regenerated CM400(1:2). Further, the regenerated CM400(1:2) was used to adsorb phenol as mentioned above.

## 3. Results and Discussion

### 3.1. Cellulose Hydrolysis

**Figure 1:**
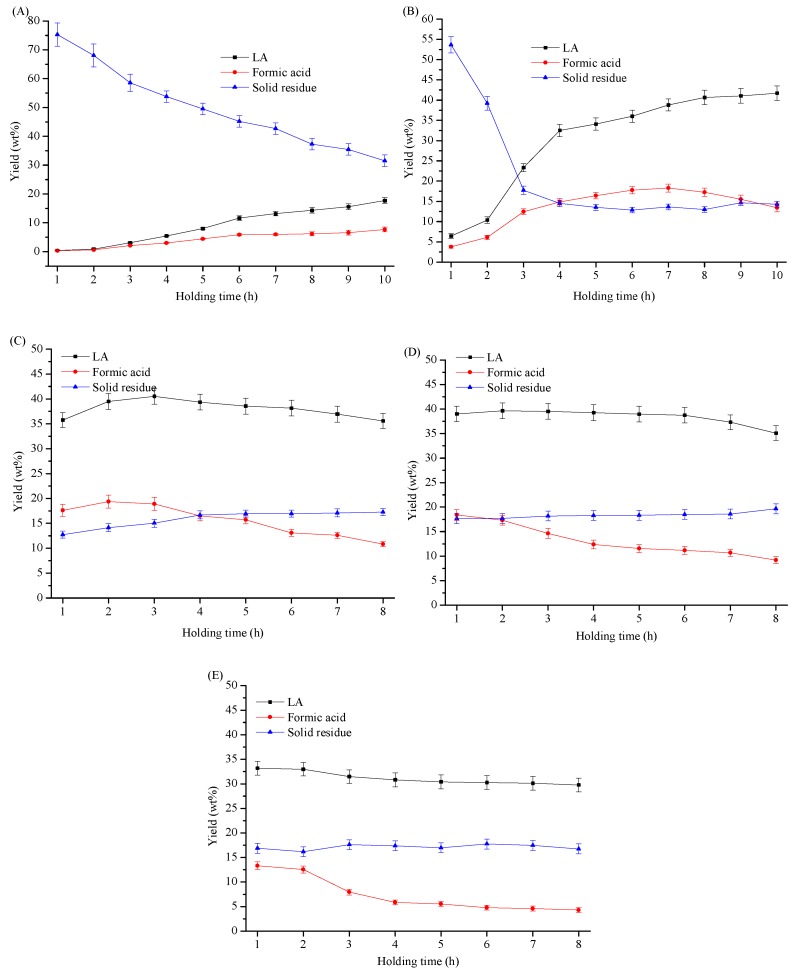
The influence of reaction temperature and time on the yield of LA, formic acid and solid residue. Reaction conditions: 0.5 g cellulose in 10 mL 0.2 M H_2_SO_4_ solution at various reaction temperatures. (**A**) 150 °C, (**B**) 165 °C, (**C**) 180 °C, (**D**) 195 °C, (**E**) 210 °C.

As shown in Figure 1, the yields of LA, formic acid, and solid residue were greatly affected by the reaction temperature and holding time of cellulose hydrolysis. It is obvious that relatively lower temperature (e.g., 150 °C) was not sufficient for LA production (yield < 20%), even with a relative long reaction time of 8 h. However, relatively higher temperature (e.g., 210 °C) is not advocated, as only a moderate LA yield (about 33 wt%) can be obtained within a relatively short time (e.g., 1 h holding time). Extension of reaction time at the higher temperature limited LA yield, due to the formation of byproducts [26,27,28]. A LA yield of around 40 wt% (or 56% of theoretical yield) was obtained at a moderate temperature of 165 °C for 8–10 h, or 180 °C for 2–3 h. This yield is relatively high as compared to the LA yield from cellulose hydrolysis, as reviewed before [4]. As compared to LA, formic acid is unstable in hydrothermal conditions [9,29], and extension of reaction time at elevated temperatures resulted in a low yield. The solid residue consisted of unconverted cellulose and/or formed humins. At a low temperature of 150 °C, the yield of the solid residue decreased with the increase of reaction time from 1 to 8 h, and the unconverted cellulose was probably the main constituent. However, at elevated temperatures (i.e., 195 °C and 210 °C), the yield of the solid residue was almost stable after 2 h of holding time, and little amount of glucose was detected in the solid residue, which indicated the complete conversion of cellulose. In the reaction solution, intermediate glucose and 5-hydroxylmethylfurfural were measured at the initial reaction stage of mild temperatures (e.g., 165 °C, 2 h holding time), while few were detected at these severer reaction conditions (i.e., 195 and 210 °C, >2 h holding time), which is consistent with former works [8]. This meant that humins was the main constituent of the solid residue at elevated temperatures of 195 °C and 210 °C with reaction time >2 h; extension of reaction time was not necessary.

Severity factor (see Equation (2)) [8,30], which has been regarded as an effective indicator to reveal the reaction severity in biomass hydrolysis, was used to evaluate the influence of reaction severity on the yield of LA, formic acid and solid residue. The severity factor combines temperature (T, °C), holding time (t, min), and pH value. Generally, a higher reaction temperature, longer reaction time and higher acid concentration (or low pH value) cause a higher severity factor. In other words, a higher severity factor means a severer reaction condition or a higher cost of production process.

Severity factor = log (t × exp((T−100)/14.75))−pH(2)

**Figure 2:**
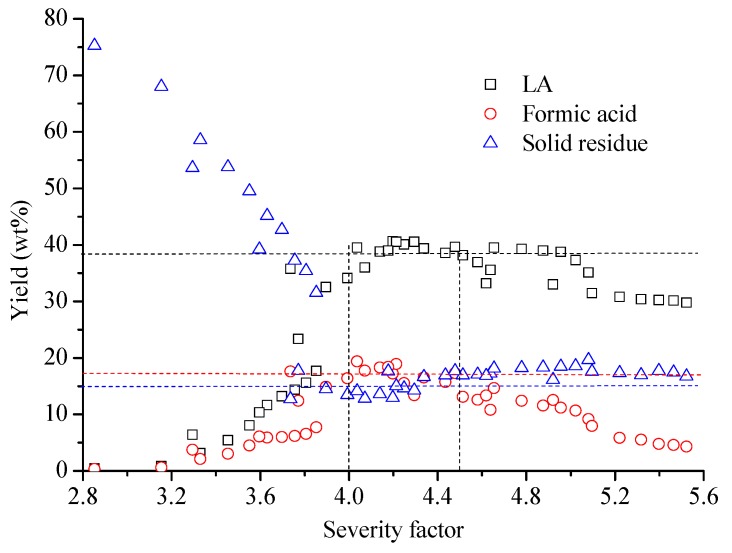
Effects of severity factor on the yield of LA, formic acid and solid residue.

The LA, formic acid and solid residue yields were 38 ± 3 wt%, 17 ± 3 wt%, and 15 ± 3 wt%, respectively, at the severity factor between 4.0 and 4.5 (see Figure 2). However, relatively low yields of LA and formic acid, and relative high yields of solid residue were detected, even with severity factor higher than 4.5 or lower than 4.0. In other words, the condition with severity factor between 4.0 and 4.5 should be preferred for cellulose hydrolysis, which might be an important reference index for further selection of industrial production conditions. A reaction condition in 0.2 M H_2_SO_4_ solution (pH = 0.4) at 180 °C for 3 h (severity factor = 4.2) was selected as an optimal condition, in which the LA, formic acid and solid residue yields were 40.5 wt%, 18.9 wt% and 15.1 wt%, respectively. The carbon content in the cellulose, LA, formic acid and solid residue were 44.4 wt%, 51.7 wt%, 26.1 wt% and 62.6 wt%, respectively. This indicated that about 47.2%, 11.1% and 21.3% of initial carbon of cellulose was transferred into LA, formic acid and solid residue, respectively. In addition, other byproducts, including water and water soluble humins, might attribute to the remaining mass yield (about 25.5 wt%). Water was formed in the creation of humins and LA by dehydration [4,8,9]. The water soluble humins were regarded as shorter and less-dense oligomers of insoluble humins, and were reported having molecular weights of about 300–500 g/mol [14,31].

### 3.2. Characterization of Solid Residue

**Figure 3:**
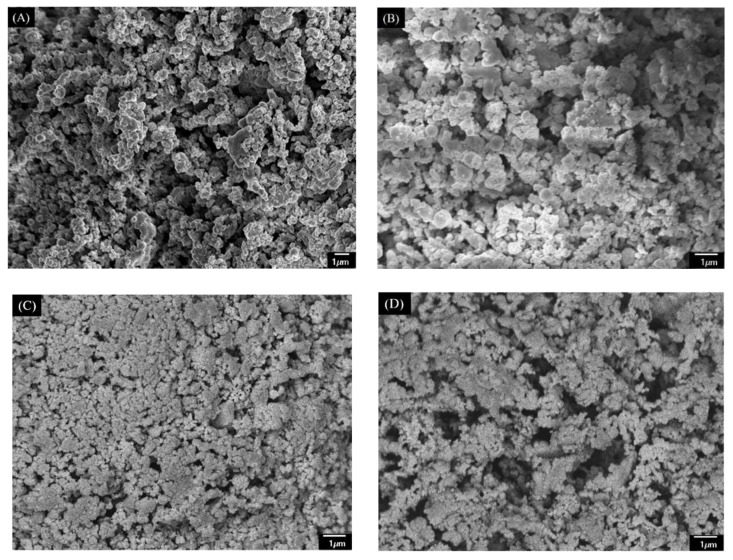
The SEM images of solid residue (**A**), CM400 (**B**), CM700(**C**) and CM900 (**D**).

**Figure 4:**
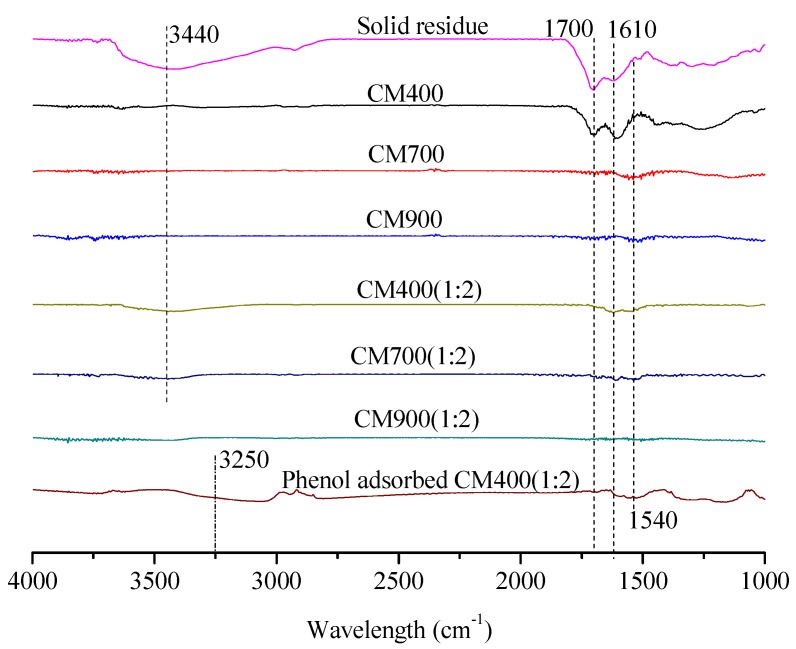
FT-IR spectra of solid residue, and various CMs.

**Figure 5:**
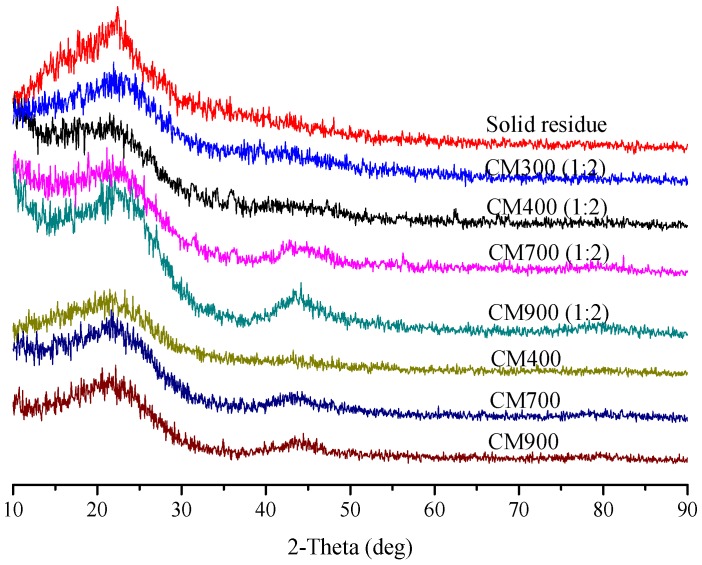
XRD spectra of solid residue and various CMs.

The solid residue obtained from the optimal condition (180 °C, 3 h) was accumulated particles with obvious clearance (see Figure 3A), which is similar to the characteristics of humins derived from glucose reported before [18,24]. In addition, the FT-IR spectrum in Figure 4 showed that the solid residue contained hydroxyl (3440 cm^−1^), methyl and methylene (2920 cm^−1^), carbonyl (1700 cm^−1^) and possible aromatic groups (1610 and 1540 cm^−1^) [32], which is also similar to that of humins [18,24,33]. The XRD spectrum in Figure 5 showed that the solid residue was amorphous carbon due to the broad diffraction peaks located at around 2θ = 22° [18,25]. The XRD spectrum confirmed the conversion of raw material cellulose owing to the disappearance of the sharp crystal peak (2θ = 22°) [34]. According to SEM, FT-IR and XRD analysis and hydrolysis discussion above, it is confirmed that the solid residue was mainly composed of humins and had similar properties to that of humins.

**Figure 6:**
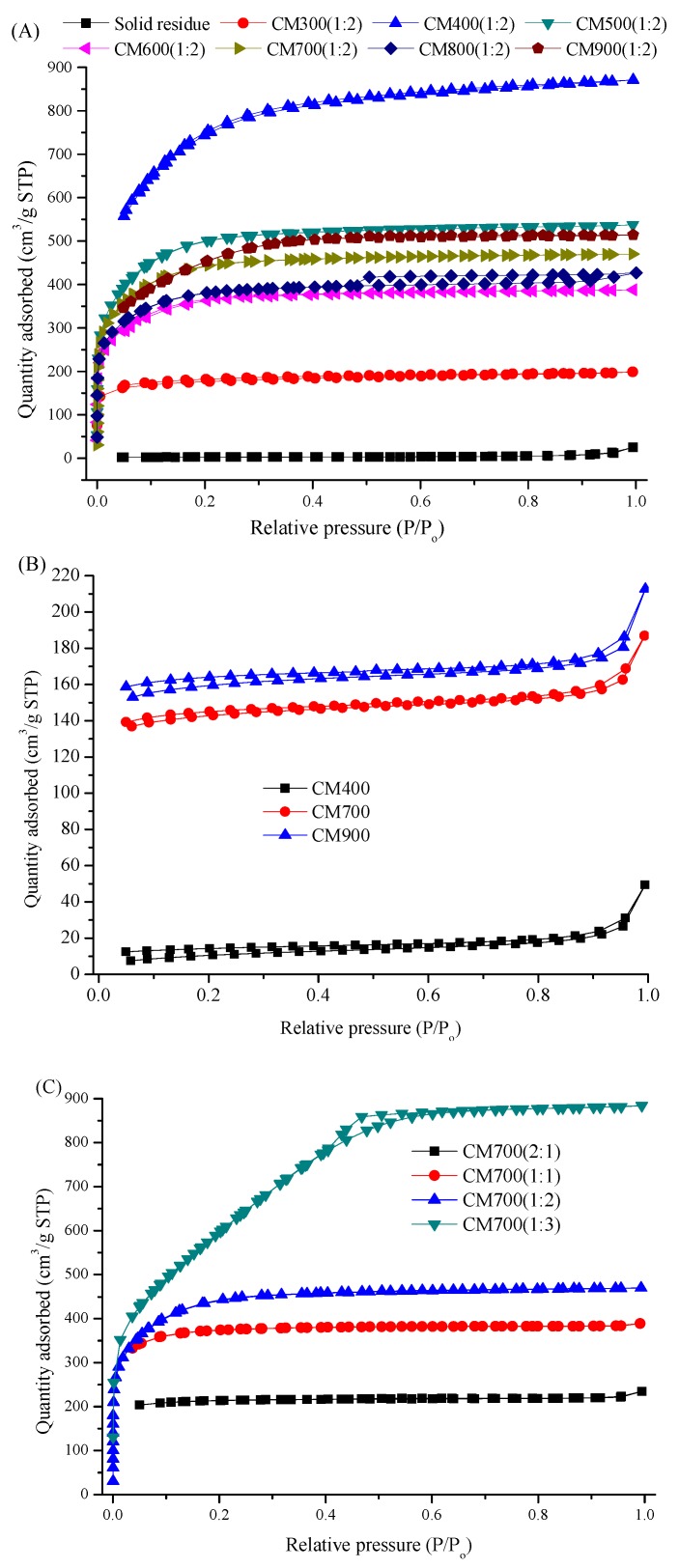
Nitrogen adsorption/desorption isotherms for various CMs at 77K. (**A**) solid residue, and CM300(1:2) - CM900(1:2); (**B**) CM400, CM700 and CM900; (**C**) CM700(2:1) – CM700(1:3).

**Figure 7:**
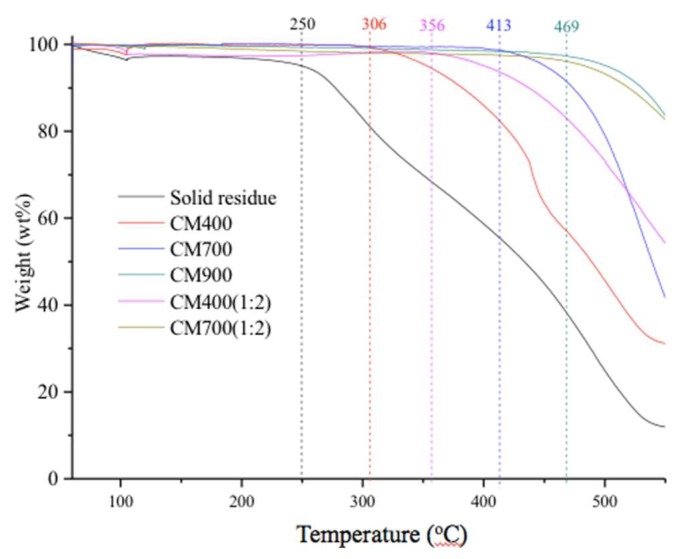
Thermal stability of solid residue, and various CMs in air.

Like humins [18], the solid residue had a very low surface area (9 m^2^/g, see Table 1), due to the quite low N_2_ adsorption capacity (Figure 6A). The TG spectra in air showed that the solid residue could be only stable below 250 °C (see Figure 7), with the evaporation of about 5 wt% moisture. When the temperature increased to higher than 250 °C, rapid weight-loss was observed, and only about 10% of the initial weight remained when the temperature reached 550 °C (see Figure 7). The low surface area and low thermal stability in air made the solid residue an insignificant candidate CM for direct application. Therefore, further pyrolysis treatments with or without ZnCl_2_ activation were conducted to convert the byproduct to profitable CM.

### 3.3. Characterization of CMs Prepared by Direct Pyrolysis

The SEM micrographs showed that the structure and morphology of the CM changed slightly with varied pyrolysis temperature (see Figure 3). From the solid residue to CM400 and to CM700, it looked like that the particles on the surface accumulated more and more closely. This indicated that expansion of particles in the solid residue occurred at 400 °C and 700 °C. However, big holes were found in the CM900, probably due to the broken of expanded particles. Pyrolysis of the solid residue led to pore formation, as high pyrolysis temperature promoted the formation of CM with high N_2_ adsorption capacity (see Figure 6B), high BET surface area and pore volume, but a low yield. As shown in Table 1, the yield of the CMs decreased obviously from 61.3 wt% of CM400 to 35.7 wt% of CM900. On the other hand, the BET surface area and pore volumes of the CM400, CM700 and CM900 were 38 m^2^/g and 0.07 cm^3^/g, 401 m^2^/g and 0.99 cm^3^/g, and 489 m^2^/g and 0.12 cm^3^/g, respectively. The thermal stability under air also increased with the increase of pyrolysis temperature, and the inflection points on the curves of CM400, CM700, and CM900 were 306 °C, 413 °C and 469 °C, respectively (see Figure 7). Pyrolysis of solid residue at these temperatures did not cause the graphitization, and these CMs were still amorphous according to XRD spectra (see Figure 5). Interestingly, the total acid density decreased while the total alkali density increased with the increase of pyrolysis temperature (see Table 1). The decrease of acid density at high temperature should be caused by the loss of acidic oxygen containing groups (i.e., hydroxyl, carbonyl and carboxyl groups), since the peaks at around 3440 cm^−1^ and 1700 cm^−1^ of the CMs produced at higher temperature had an obvious lowered intensity based on FT-IR analysis (see Figure 4).

### 3.4. Characterization of CMs Prepared with ZnCl_2_ Activation

In comparison to the CMs prepared by direct pyrolysis and pyrolysis after ZnCl_2_ activation (see Figure 4 and Figure 5), ZnCl_2_ activation did not change the amorphous carbon characteristics and the distribution of functional groups. Similar change trends of thermal stability, and acid/alkali density with pyrolysis temperature were also found for the all CMs prepared, no matter whether ZnCl_2_ activation was conducted or not (see Figure 7 and Table 1). However, the thermal stability of CM400(1:2) and CM700(1:2) was better than that of CM400 and CM700, respectively (see Figure 7), indicating that the ZnCl_2_ activation promoted the formation of CM with a more stable structure. Figure 6A showed N_2_ adsorption isotherms of CM300(1:2)–CM900(1:2), all of which exhibited isotherms of type I according to the International Union of Pure and Applied Chemistry (IUPAC) classification. Sharp curves and inflection points were observed at low-pressure region (P/P_0_ < 0.1), and the low-pressure region contributed a major N_2_ adsorption capacity, indicating a wide distribution of pores with small size.

**Figure 8:**
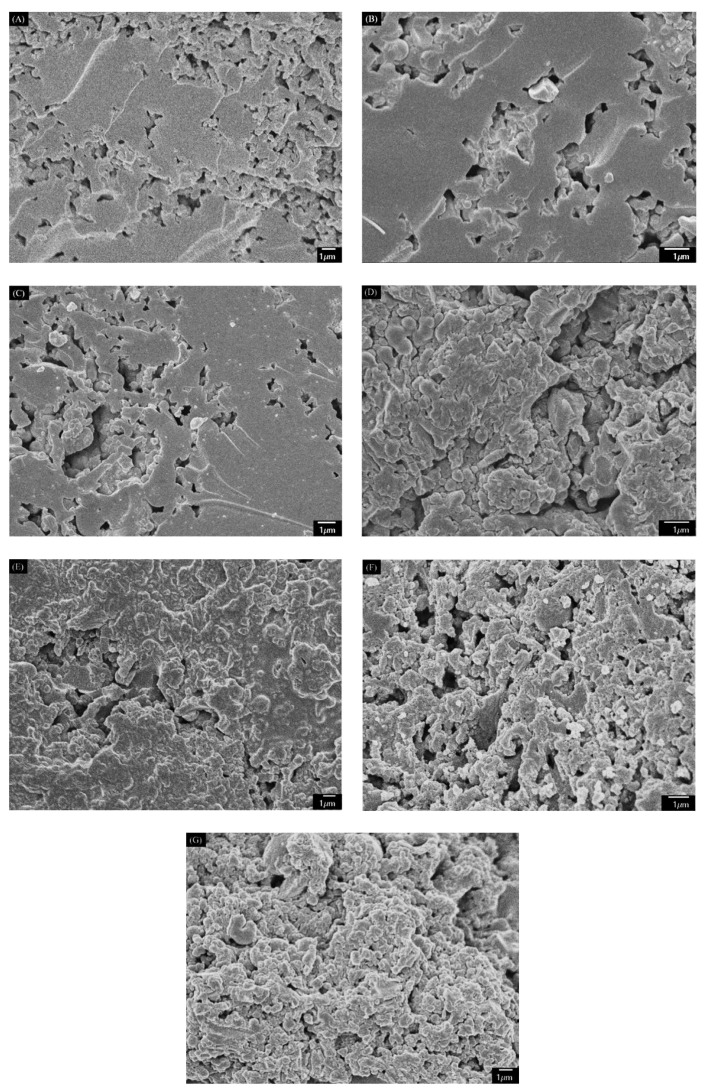
SEM images of CMs from ZnCl_2_ activation. (**A**) CM300(1:2); (**B**) CM400(1:2); (**C**) CM500(1:2); (**D**) CM600(1:2); (**E**) CM700(1:2); (**F**) CM800(1:2); (**G**) CM900(1:2).

The promotion of structural transformation and pore formation by ZnCl_2_ activation was examined by SEM, and BET surface area/pore volume analysis (Table 1). The SEM images showed that ZnCl_2_ indeed affected CM surface topography, as the CM300(1:2)–CM900(1:2) had smoother surface structures than the solid residue, CM400, CM700 and CM900 (see Figure 3 and Figure 8). This was probably caused by the impregnation and swelling effect of ZnCl_2_ in the activation process [35], which filled and leveled up clearance. Pyrolysis temperature was another factor affecting the surface topography for production of CM300(1:2)–CM900(1:2) (see Figure 8). The CM300(1:2)–CM500(1:2) had smooth flat surfaces, in which corroded porous structures were observed. On CM600(1:2) and CM700(1:2), however, the smooth flat structure was somewhat destroyed, and the corroded holes decreased. Further increase of the pyrolysis temperature formed CM800(1:2) and CM900(1:2), and a more serious destruction of the flat surfaces was found.

The formation of pores was also affected by the combined actions of ZnCl_2_ and pyrolysis. As shown in Table 1, with the increase of pyrolysis temperature, the BET surface area increased from 300 °C (532 m^2^/g) to 400 °C (2510 m^2^/g), decreased from 400 °C to 600 °C (1184 m^2^/g), and then increased from 600 °C to 900 °C (1526 m^2^/g). A similar trend of total pore volumes was found, as the total pore volumes of CM300(1:2), CM400(1:2), CM600(1:2) and CM900(1:2) were 0.092, 1.02, 0.30 and 0.61 cm^3^/g, respectively. The BET surface and pore volume of CM400(1:2) were almost 66.6 and 14.6 times those of CM400, respectively. Therefore, ZnCl_2_ activation played a major role in the formation of pores. However, the BET surface and pore volume of CM700(1:2) decreased to 3.6 and 3.7 times those of CM700, while the BET surface and pore volume of CM900(1:2) further decreased to only 3.1 and 5.1 times those of CM900, respectively. At these high temperatures of 700 °C and 900 °C, the effects of ZnCl_2_ became weak, and both high temperature pyrolysis and ZnCl_2_ activation promoted pore formation. The low activation effect at high temperature was probably due to the volatilization of ZnCl_2_, which has a boiling point of 732 °C [36].

Dosage of ZnCl_2_ is another important factor affecting the formation of pores. A high dosage of ZnCl_2_ promoted pore formation according to N_2_ adsorption isotherms shown in Figure 6C, and the data of surface area and pore volume listed in Table 1. The BET surface area and pore volume of CM700, CM700(2:1), CM700(1:1), CM700(1:2) and CM700(1:3) were 390.5 m^2^/g and 0.10 cm^3^/g, 625 m^2^/g and 0.10 cm^3^/g, 1130 m^2^/g and 0.13 cm^3^/g, and 2202 m^2^/g and 1.42 cm^3^/g, respectively. However, a high dosage of ZnCl_2_ sacrificed the CM yield. The yield of CM700 was 49.7 wt%, while the yield of CM700 (1:3) decreased to 21.4 wt%.

### 3.5. Application of CMs for Phenol Adsorption

Phenol is one of the most-used model contaminants for adsorption studies [18], owing to the production of huge phenolic wastewater in modern industry; therefore, a series of the CMs were used to adsorb phenol in aqueous solution. CM400(1:2) was selected as a typical sample for the adsorption due to its highest BET surface area and good yield. The adsorption of phenol on CM400(1:2) was directly reflected by the FT-IR spectra, as shown in Figure 4. There were much stronger peaks on the phenol adsorbed CM400(1:2) than the CM400(1:2) at 3250 cm^−1^, and 1610 and 1540 cm^−1^, due to the hydroxyl and benzene ring of phenol, respectively. This was also confirmed by the decrease of BET surface area and pore volume to 1781 m^2^/g and 0.69 cm^3^/g, respectively, after the phenol adsorption on the CM400(1:2) (see Table 1).

**Figure 9:**
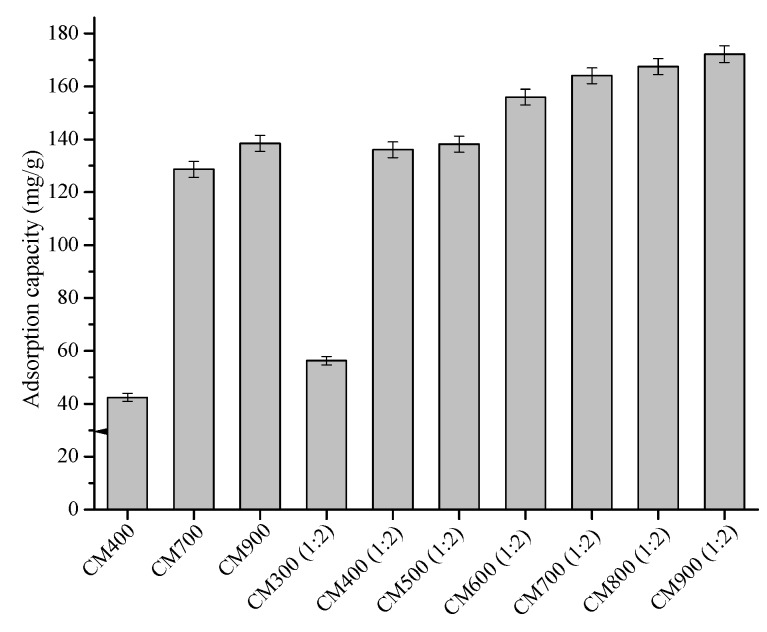
Adsorption capacity of phenol on various CMs.

**Figure 10:**
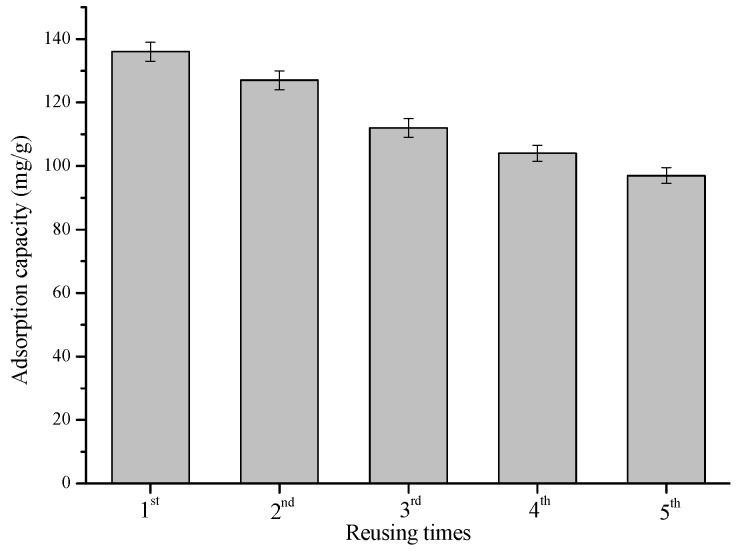
Effects of reusing times of CM400(1:2) on phenol adsorption.

As shown in Figure 9, the phenol adsorption capacity of the CMs increased with the pyrolysis temperature, with or without ZnCl_2_ activation. For example, the adsorption capacity of CM400 was only 42.4 mg/g, while that of CM900 reached 138.4 mg/g. Compared with the CMs produced by direct pyrolysis, the CMs produced by ZnCl_2_ activation showed obvious improved phenol adsorption capacity. For example, CM400(1:2) had a phenol adsorption capacity of 136.0 mg/g, which was 3.0 times that of CM400. One explanation for the improved adsorption capacity is the increased BET surface area and pore volume, as discussed above. However, CM900(1:2) had a lower BET surface area but higher phenol adsorption capacity than CM400(1:2). This meant that the BET surface area and pore volume should not be the only factor affecting phenol adsorption. The total alkaline sites (see Table 1), increased with the increase of pyrolysis temperature, would promote the adsorption of acidic phenol by π–π dispersion force [18,37], should be the other reason for phenol adsorption. Importantly, the phenol adsorption capacity (136–172 mg/g) of CM400(1:2)–CM900(1:2) and CM900 are comparable to that of activated carbons reported before [38,39,40,41]. In addition, the used CM400(1:2) can be simply regenerated after a simple methanol Soxhlet extraction process. The regenerated CM400(1:2) exhibited a comparable adsorption capacity of 97 mg/g even in the 5th reusing cycle (see Figure 10). Therefore, the CMs obtained from pyrolysis, especially with ZnCl_2_ activation are potential adsorption materials due to excellent adsorption capacity, good reusability and cheap raw materials (waste hydrolysis residue).

On considering the yield, surface area and pore volume, phenol adsorption capacity, and temperature required for pyrolysis, CM400(1:2) is a preferred product of the solid residue. Thus, in a preferred two-step reaction process, i.e., hydrolysis at 180 °C for 3 h and pyrolysis at 400 °C with ZnCl_2_ activation, 100 g cellulose was finally converted to 40.5 g LA, 18.9 g formic acid, and 8.5 g carbon adsorption material. In addition, CM400(1:2) had a carbon content of 84.2 wt%. Therefore, about 74.4% of the initial carbon of cellulose was transferred into value-added products, i.e., 47.2% carbon in LA, 11.1% carbon in FA, and 16.1% carbon in CM400(1:2).

## 4. Conclusions

In this work, a sequential two-step process was developed to maximize the utilization of cellulose, including the acid hydrolysis of cellulose for LA production and pyrolysis of hydrolysis residue for CM production. In the hydrolysis step, a reaction condition with a severity factor between 4.0 and 4.5 was preferred, in which the yield of LA, formic acid and solid residue reached 38 ± 3 wt%, 17 ± 3 wt%, and 15 ± 3 wt%, respectively. In the pyrolysis step, ZnCl_2_ activation was recommended, which promoted the formation of CMs with high BET surface area, pore volume and phenol adsorption capacity. CM400(1:2) was a preferred product with a BET surface area and phenol adsorption capacity of 2510 m^2^/g and 136 mg/g, respectively, and the used CM400(1:2) could be simply regenerated by methanol Soxhlet extraction method. Owing to its excellent adsorption capacity and good reusability, CM400(1:2) could be used as a promising CM for wastewater treatment. In a preferred two-step process, the final yield of LA, formic acid and CM400(1:2) was 40.5 wt%, 18.9% and 8.5 wt%, respectively. Importantly, the total carbon utilization ratio of the three value-added products reached 74.4%, which makes the sequential two-step process a promising technology for the biorefinery industry.

## Figures and Tables

**Table 1:** Properties of solid residue and various carbon materials.

Samples	Yield (%)	Surface Area (m^2^/g)	BJH Adsorption Pore Volume (cm^3^/g)	Total Acidic Contents (mmol/g)	Alkali (mmol/g)
Solid residue	-	9.0	0.02	-	-
CM400	61.3	37.7	0.07	1.4	0.25
CM700	49.7	390.5	0.10	1.1	0.69
CM900	35.7	488.8	0.12	0.5	0.74
CM300(1:2)	69.1	532	0.092	1.4	0.20
CM400(1:2)	56.1	2510	1.02	1.4	0.32
CM500(1:2)	50.5	1594	0.44	1.2	0.69
CM600(1:2)	44.7	1184	0.30	1.1	0.75
CM700(1:2)	38.5	1392	0.37	0.9	0.80
CM800(1:2)	34.7	1440	0.59	0.9	0.85
CM900(1:2)	30.6	1526	0.61	0.8	0.83
CM700(2:1)	46.1	625	0.1	1.2	0.71
CM700(1:1)	41.2	1130	0.13	1.1	0.77
CM700(1:3)	21.4	2202	1.42	0.9	0.89
Phenol adsorbed CM400(1:2)	-	1781	0.69	-	-
